# The Notch Pathway in Breast Cancer Progression

**DOI:** 10.1155/2018/2415489

**Published:** 2018-07-08

**Authors:** Emmanuel N. Kontomanolis, Sofia Kalagasidou, Stamatia Pouliliou, Xanthoula Anthoulaki, Nikolaos Georgiou, Valentinos Papamanolis, Zacharias N. Fasoulakis

**Affiliations:** ^1^Department of Obstetrics and Gynecology, Democritus University of Thrace, 681 00 Alexandroupolis, Greece; ^2^Department of Obstetrics and Gynecology, Bodosakio General Hospital of Ptolemaida, 502 00 Ptolemaida, Greece; ^3^Department of Radiotherapy/Oncology, Democritus University of Thrace, 681 00 Alexandroupolis, Greece; ^4^Stoke Mandeville & Wycombe General Hospital, Buckinghamshire NHS Trust, UK; ^5^Department of Obstetrics & Gynecology, General Hospital of Korinthos, 201 00 Korinthos, Greece

## Abstract

**Objective:**

Notch signaling pathway is a vital parameter of the mammalian vascular system. In this review, the authors summarize the current knowledge about the impact of the Notch signaling pathway in breast cancer progression and the therapeutic role of Notch's inhibition.

**Methods:**

The available literature in MEDLINE, PubMed, and Scopus, regarding the role of the Notch pathway in breast cancer progression was searched for related articles from about 1973 to 2017 including terms such as “Notch,” “Breast Cancer,” and “Angiogenesis.”* Results*. Notch signaling controls the differentiation of breast epithelial cells during normal development. Studies confirm that the Notch pathway has a major participation in breast cancer progression through overexpression and/or abnormal genetic type expression of the notch receptors and ligands that determine angiogenesis. The cross-talk of Notch and estrogens, the effect of Notch in breast cancer stem cells formation, and the dependable Notch overexpression during breast tumorigenesis have been studied enough and undoubtedly linked to breast cancer development. The already applied therapeutic inhibition of Notch for breast cancer can drastically change the course of the disease.

**Conclusion:**

Current data prove that Notch pathway has a major participation and multiple roles during breast tumor progression. Inhibition of Notch receptors and ligands provides innovative therapeutic results and could become the therapy of choice in the next few years, even though further research is needed to reach safe conclusions.

## 1. Introduction

Breast cancer represents the leading diagnosed cancer in the United States and the second main cause of cancer death in America, which accounts for approximately 30% of all new cases of cancer diagnosed in female patients [1]. Age, early menarche, late menopause, obesity in postmenopausal women, high concentration of endogenous estrogen, and heredity are the main predisposing factors for developing breast cancer, while recently there has been growing interest in the Notch signaling pathway and its role in breast cancer.

Over the past decades, Notch was identified as oncogene in mouse mammary tumor virus-infected mice. Studies revealed that aberrant Notch signaling induces mammary gland carcinoma in transgenic mice and, a few years later, Notch was also detected in human breast cancer predisposing to play a major role in breast cancer development due to its participation in cell proliferation, differentiation, and apoptosis [2]. Since then, multiple hypotheses were suggested about Notch signaling pathway in breast cancer progression. To date, even though some aspects remain unclear, the role of Notch in breast cancer is being clarified, revealing a special participation of each of the Notch receptors and ligands in breast cancer development.

The aim of this review is to identify, summarize, and analyze current knowledge considering the main role and the therapeutic inhibition of the Notch signaling pathway in breast cancer progression.

## 2. Breast Cancer

Over the past decades, breast cancer vigilance has surmounted both the medical profession and the general population. Counseling, an in-depth discussion, risk evaluation, and clinical assessment for strong candidates to develop breast cancer are complex and essential. In 2018, 1,735,350 new cancer cases and 609,640 cancer deaths are projected to occur in the United States [3]. The risk of developing breast cancer varies according to tumor subtype; however, genetic predisposition is responsible for 5% to 10% of all breast cancer types [4].

The involvement of the axillary lymph nodes is the single most reliable indicator of prognosis [5]. Higher incidence of bilateral breast disease, family history, earlier age onset of the disease, genetic predisposing potential, and reporting of other neoplasms in other family members are parameters that differentiate the sporadic from the inherited breast cancer. One in 400 women is found to be positive to BRCA mutation and the average cumulative cancer risk in those women by the age of 70 years ranges from 45% to 65% for breast cancer [6].

A positive family history is a strong risk factor for cancer-related mortality in breast carcinogenesis [7]. Moreover, additional risk factors are women with reported early menarche and late menopause, a small number of gestations and short breast-feeding periods, unhealthy nutritional behaviors, smoking or exposure to radiation, and clinically determined atypical hyperplasia. Hormone replacement therapy (HRT), increased alcohol and fat consumption, nutrition low in fruit and vegetables, bottle versus breast-feeding, use of contraceptive pills, and induced abortions have not yet been clarified in terms of either increasing or decreasing the incidence of breast neoplasms [8–10]. Whether screening and diagnosis play a role in reducing mammary gland mortality has also been questioned and until today many studies focus on providing a clear answer [11, 12].

## 3. Breast Cancer Molecular Subtypes

According to the American Cancer Association [13], there are currently four major breast tumor molecular subtypes with prognostic differences on patients outcome based on expression or not of specific biological markers: estrogen receptors (ER), progesterone receptors (PR), and human epidermal growth factor receptor 2 (HER2) [Table 1].

Luminal A accounts for about 40% of breast cancers and represents the most common subtype. These tumors are usually hormone-receptor positive (ER+ and/or PR+) and HER2 negative. Clinical characteristics of Luminal A subtypes reveal high survival rates; they are less aggressive tumors with low recurrence rates (low Ki67) than the other subtypes and have the most favorable short-term prognosis due to favorable response to endocrine therapy [14].

Luminal B are also hormone-receptor positive (ER+ and/or PR+) and HER2+/- tumors, with lower prevalence than Luminal A but with worse prognosis. They represent 10-20% of breast cancers. They have high proliferation rates (high Ki67) but usually respond to endocrine therapy [15].

Basal-like types are tumors defined by the absence of all hormone receptors (ER-, PR-, and HER2-) and represent 10-20% of breast cancers. These triple negative breast cancers (TNBC) are aggressive, with high histologic grate, high rates of distant metastasis after surgery, and poor short-term prognosis due to lack of targeted therapies with specific activity [16].

HER2 enriched represent hormone receptor negative (ER-, PR-) and HER2 positive tumors. They tend to be high grade, node positive, aggressive tumors, with poor survival rate and represent 10% of breast cancers. In contrast to basal-like tumors, targeted therapies exist [17].

## 4. VEGF and Notch: The Two Prevailing Mechanisms in Tip and Stalk Cell Evolvement

The Vascular Endothelial Growth Factor (VEGF) and the Notch families establish an important principle in normal vessel growth and the identification that Notch signaling pathway has a major impact on arterial specification, sprouting angiogenesis, and vessel maturation led us to a better understanding of vessel specialization and tumor vascularization.

### 4.1. The VEGF Pathway

The VEGF pathway is a highly conserved signaling system applied in vessel and tissue growth that, in cooperation with the Notch system, both engage in the regulation of vessel formation and sprouting. The pathway encompasses six ligands (VEGF-A, VEGF-B, VEGF-C, VEGF-D, VEGF-E, and PIGF) with three cognate receptors (VEGFR-1, VEGFR-2, and VEGFR-3) [18, 19]. Blood vessels are influenced by the expression of these factors that cause the endothelial cells to migrate towards the tip or stay in the luminal region of a blood vessel to guarantee the stability and perfusion of the vascular network [18, 20].

### 4.2. The Notch Signaling Pathway

**Figure 1:**
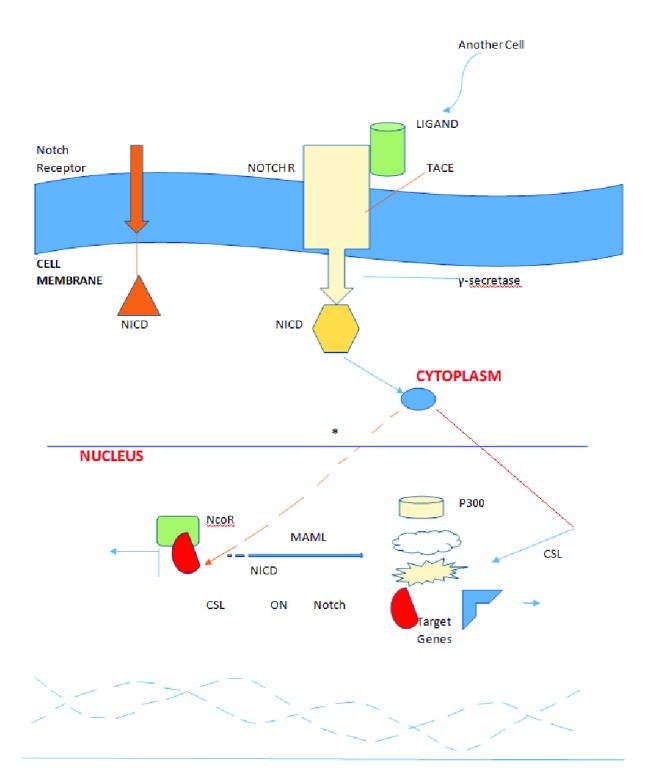
The Notch signaling pathway. ^*^NICD dissociated NcoR from CSL and CSL becomes the activating agent that attracts other complexes (P300, MAML).

The Notch pathway describes the interaction between two adjacent cells, whereas the first cell is carrying a ligand and the second is carrying a receptor programmed to combine with the ligand. Four heterodimeric transmembrane Notch receptors (1-4) that react with the transmembrane ligands Delta-like (Dll) 1,2,4 and Jagged (JAG) 1 and 2 are identified in humans. The Notch receptor is activated by binding to a ligand presented by the neighboring cell and activates Notch signaling with the ligand moving towards the receptor. The signal is rapidly passed on from the cell membrane to the nucleus and the adjoining cells; this reaction triggers a series of biochemical events (proteolytic cleavages on the receptor) which can affect the expression of genes. Notch is split with the intracellular domain (NICD) discharged within the cytoplasmic region, travelling and eventually penetrating the nuclear membrane. In the nucleus, there is a reaction between the NICD and the transcription factor CSL (mammalian CBF-1), which causes the emerging of a transcriptional activation complex and the simultaneous expression of downstream target genes (Hes and Hey family clusters) (Figure 1).

### 4.3. The “Tip” and “Stalk” Cell Selection

Notch signaling controls a cohort of genes involved in angiogenesis, including the VEGF receptors. Newly formed blood vessels are formed of “tip and stalk” cells, cells that acquire a specific route with deadly accuracy. These cells are identified by a specific gene expression pattern besides their structural and functional dissimilarities. The “tip” and the “stalk” cells compose the two major types of the endothelial cells; the “tip” cell is based at the far end leading the vessel in an outward growing mode and expresses high levels of VEGFR-2, VEGFR-3, and platelet-derived growth factor-B, while the “stalk” cell is located adjacent to tip cell, expressing high levels of VEGFR-1 and proliferating in the lumen of the newly formed vessel. In newly formed sprouts, Notch system conducts these cells in the lumen of the newly formed vessels, distinguishing the endothelial “stalk” cells that will form the final capillary.

Cells with stronger VEGF receptor-2 (VEGFR-2) and VEGFR-3/Flt-4 signature feature overexpress Dll-4 and promote a tip cell fate during vessel sprouting. The Notch pathway is being activated by Dll-4 in neighboring endothelial cells (ECs) and restricts the expression of VEGFR-2 and VEGFR-3, repressing the tip cell type and consequently urging towards the stalk cell. With an inactive Notch signaling system, ECs join to form extensive vessel networks, the blood, and lymphatic vasculature in response to the presence and action of VEGF. The vascular network predisposes the presence and multiplication of endothelial and mural cells and their strict migration and connectivity to form sprouts that will eventually construct functional arteries, capillaries, veins, and lymphatics. The Notch ligands are expressed in response to high expression of VEGF ligands determining the beginning of sprouting. Malfunction of Notch signaling pushes these cells away from the vessel and towards the tip region of the blood vessels [19].

### 4.4. Tip and Stalk in Breast Cancer

The destiny of the tip and stalk cells is regulated as mentioned by VEGF and Notch signaling. The aim of this process is the final development of a capillary network that can support the growth of organs, the immune system, a constant body temperature, and the fine regulation of the homeostatic internal system during life [20–23].

Cancer invasion follows a common specific pattern of steps, including cell proliferation, angiogenesis, and distant organ adherence. The environment in solid tumors is oxygen-deficient; cells require oxygen and nutrients in order to survive. Tumor growth is dependent on angiogenesis, a biological procedure that relies on the expression of proangiogenic factors (e.g., VEGF-A and Dll-4), as well as the “tip and stalk” procedure.

Clinical studies have identified that the first solid tumor in which Notch was implicated was breast cancer [24]. Through year, deregulation of the normal-canonical Notch pathway has been proven to participate actively in cancer development. The uncontrolled cell multiplication, the impairment of the programmed cell death mechanism, and the exponential cellular growth are the basic features of tumor growth [25, 26]. Dll-4 is found in the region of the endothelial cells participating in angiogenesis via “tip and stalk” procedure, obeying VEGF signals, and is found to be overexpressed in breast cancer [27].

As tumors grow, they will eventually reach a size where they will require additional vasculature to sustain constant advancement of the newly formed cancer tissue. The traditional organized structure of arterioles, capillaries, and venules is abolished. Neoplastic vessels are defective and acquire an abnormal contour, are dilated, form shunts, follow an irregular course, and are devoid of pericytes [28–31]. They are characterized by rapid multiplication, inadequate blood circulation, hemorrhagic areas, evident permeability, and disordered spreading of the pericytes population [32, 33]. The appearance of abnormal vessels and the insufficient tumor growth are also related to the correlation between the Dll-4 expression and the VEGF molecule in “tip” and “stalk” cell; blockade of the VEGF action on neoplastic structure downregulates Dll-4 expression [21, 22, 34]. Notch-1 and Notch-4 receptors accompanied by their cognate ligand Dll-4 are active regulators of the preliminary vascular development. The endothelial tip cells accumulate vast quantities of the Delta-like ligand 4 and the stalk cells on the other hand possess a different ligand, the Jagged-1 protein molecule within the cellular lumen. The coexistence and antagonism of Delta-like 4 ligand and Jagged-1 strengthen Notch signaling, regulate VEGF titers and EC multiplication, and stabilize vasculature [21–23, 34, 35].

Due to their particular role, tip cells become more and more of great interest as potential target for antiangiogenic therapies. Inhibitors for retinal tip cells (platelet-derived growth factor) have already been proven to effectively block tumor angiogenesis, giving promises of great medical achievements in the next decades [36].

## 5. Τhe Notch Activating System in Breast Cancer

The Notch signaling system is the critical fundamental pathway, a unique cellular program that is encountered in cell type specification and organ development. It is the main regulator of cell destination and differentiation. Notch expression is regulated by hypoxia and inflammatory cytokines (IL-1, IL-6, and leptin) and activated by ligand binding. Any type of proangiogenic signal can affect endothelial cells to evolve into either the tip or the stalk cellular norm. It is identified by four Notch receptors, Notch-1, Notch-2, Notch-3, and Notch-4; there are five related ligands, Jagged-1, Jagged-2, (equivalent to Serrate), and Delta-like-1 (Dll-1), Dll-3, and Dll-4; these protein molecules are all membrane-bound, conducting paracrine signals and existing on arterial and not venal vessels [18–20]. Notch signaling depends solely on the binding of the Notch receptor and the Delta/Serrate ligand through endocytosis and the cell-to-cell communication process. Tumors acquiring a high expression profile of Notch ligands are an undisputable fact that relates them to their aggressive clinical behavior. The Dll-4 protein molecule is encountered in physiological and pathological angiogenesis; Dll-4 protein serves as a negative regulator in tumorigenesis and as a positive regulator in tumor progression; the explanation of this duality of action still remains a mystery. The presence and expression of Dll-4 in neoplastic cells result in restriction of new blood vessel development, while they simultaneously trigger the formation of large diameter blood vessels with adequate perfusion and oxygenation capability. Notwithstanding the increased angiogenesis, tumor growth and expansion are meager within the solid mass; this is due to the fact that the novel vessel system is nonfunctional [18–21].

The starting point for Dll-4 upregulation is the arterial endothelial luminal region. Upregulation of Dll-4 ligand in the cardiovascular system guides new vessel sprouting. Deregulation of the Dll-4 ligand has a negative impact on vessel formation; new vessels are indeed being constructed but their functional ability is restricted; this fact results in impaired tumor growth. Downregulation of the Dll-4 ligand results in appearance of novel nonfunctional vasculature in neoplasms. This nonfunctional vasculature is totally incapable of readily conveying blood to the neoplasm and results in minor tumor growth. Lateral inhibition, boundary formation, and lineage decision compose the different types of cell-to-cell communication [28, 31].

## 6. Notch and Breast Cancer Subtypes 

Cell destiny determination and tumorigenesis are pathways led by Notch signaling, while the four Notch receptors acquire different roles in cancer development [37–40]. There have been multiple studies revealing association of high levels of Notch signaling with different subtypes of breast cancer. The aberrant activation of the Notch signaling has been reported to be present and implicated in human cancer pathogenesis many times over the past decade, acting either positively or negatively to patients' outcome. On the contrary, negative regulators of the Notch pathway, such as Numb, reveal implication due to absence in even 50% of breast cancer cases [41–44].

There is accumulating evidence that underlines the direct involvement of the Notch pathway in normal mammary gland growth and breast carcinogenesis [42, 45–47]. Breast cancer clinical outcome was accompanied by high expression of Notch-1,-3,-4 pathways, and Notch-2 was identified as a tumor suppressor in many studies [48–52]. Evidence proving the fundamental role of Notch system in breast carcinogenesis comes from the mouse-mammary tumor virus (MMTV), where Notch-1 and Notch-4 genes have been detected [45, 53–56]. The MMTV belongs to the retroviruses family and induces the growth of mammary tumors on mice through the process of insertional mutagenesis (mutagenesis of DNA by the insertion of one or more bases that can occur naturally mediated by virus or transposon or can be artificially created for research purposes in the lab). The genes Notch-1 and Notch-4 are primary targets for the MMTV. Through the processes of insertion and rearrangement, mutations are being created, which induce epithelial mammary oncogenesis.

There are actually two genes, Notch-1 and Notch-4, responsible for tumorigenesis [56]. The int-3/Notch-4 oncoprotein converts the mammary epithelial cells to neoplastic cells and induces the development of adenocarcinoma in mice. Approximately 20% of these tumors have been injected with the mouse mammary tumor virus provirus directly in the Notch-4/int-3 locus. Following this step, analogous mouse mammary insertions-type has been illustrated in the laboratory. Notch-1 is the second gene participating through proviral insertion in mammary neoplasms in mice.

A large number of experiments on animal models and Notch genes have been conducted in contrast to human breast cancer genetics, where clinical information is limited [57, 58]. The Her-2 and Wnt pathways are two examples of genetic systems involved in breast tissue tumor growth [59]. The Wnt pathway plays a role in the ventral and dorsal axial development in the upper limb and the mammary gland. Aberrant functioning of the Wnt signaling system results in breast tumor appearance [60, 61]. The Wnt genes Lef-1 and Axin-2 in cooperation with the Notch ligands Dll-3 and Dll-4 were detected in an array of 34 breast neoplasms, indicating that a type of carcinogenesis is on the way [62]. Mammary gland development and carcinogenesis on the same organ depend on Notch and Wnt signaling pathways. The Wnt system restricts mammary gland growth and differentiation of the breast tissue during gestation and lactation with its 19 Wnt genes, 10 Frizzled (Fzd) receptors, and 2 low-density lipoprotein receptor-related protein (LPR) coreceptors [60–62].

### 6.1. Luminal Breast Cancer

Luminal breast cancer represents the most common breast cancer type (>70%), with favorable prognosis that responds well to antihormonal therapy [63]. Dou et al. recently reported (2017) the Notch-3 receptor prevailing in Luminal A breast cancer subtypes via transactivating ER*α* expression in breast cancer [64, 65].

Touplikioti et al. published in 2012 a study where the authors examined the expression of Notch receptors in different subtypes of 200 human breast cancers. According to their results, ER(+) and/or PR(+) combined to HER2(-) (Luminal A or B subtype) mainly reveal high expression of Notch-4 but revealed, without statistical significance, low levels of Notch-1 and -3 [66].

### 6.2. Basal-Like Breast Cancer

Speiser et al. (2012) studied the expression of Notch-1-4 receptors on basal-like/TNBC [67]. TNBC is a heterogeneous neoplastic disease, considered to be the most devastating type of breast cancer, characterized as mentioned by complete lack of hormone receptors and the human epidermal growth factor receptor 2 (HER-2/neu) [68]. Higher Notch-1 activity was observed in TNBC credited in the PEST domain mutation identified in Notch-1. In patient-derived cancer xenografts with Notch-1 mutation, the detection of high sensitivity of a gamma secretase inhibitor (gamma secretase is responsible for activation of Notch receptors) confirmed the results [64, 69, 70].

Touplikioti et al. published in 2012 a study also reporting the relation of TNBC with high expression of Notch-1, additionally indicating that Notch-3, a receptor associated with vessel formation, is also expressed in this tumor type because of the increased angiogenesis that TNBC required. On the contrary, Dickson et al. reported the detection of Jagged-1 and Notch-4 mRNAs in high titers in breast cancer patients with clinical poor prognosis including TNBC subjects. Owing to total absence of receptors (estrogen, progesterone, and human epidermal growth factor receptors), there is no definitive and targeted therapy in TNBC [42, 68].

## 7. Clinical Research on the Expression of Notch Signaling

Clinical research on Notch receptors revealed the entire molecular profile; Notch-1 is in the heart tissue and the vascular endothelium and Notch-4 is strictly and to a greater extent detected in vascular endothelial cells (ECs). Notch-3 is part of the vascular smooth muscle cell network. Dll-4 is believed to be the designated ligand for Notch-1 and -4 during the very early stages of vessel development. Stalk cells demonstrate the Jagged-1 ligand and tip cells strongly express Dll-4. Jagged-1 is also present in smooth muscle cells surrounding the arteries and plays an important role in their maturation procedure [71].

Among the Notch receptors, Notch-3 and Notch-4 seem to have eminent role in normal breast growth, even though Notch-4 demonstrates increased activity (upregulation) in the neoplastic tumor vasculature [72, 73]. Nevertheless, the vascular network density does not seem to be affected by the presence of the Notch-4 receptor. It is well established that tumor angiogenesis is dependent on vessel sprouting. Studies found that, in Notch-3 expression, the luminal-type colonies were structured at a low rate, while the myoepithelial-type conglomerates were increased. Upregulation of the active form of Notch-4 suppressed the differentiation step of the typical breast epithelial cells [74–76].

More recently, studies reported that high expression level of Notch-3 NICD in breast tissue is related to incidence of mammary gland neoplasms, indicating that the receptor has oncogenic potential. Yamaguchi et al. (2008) showed that Notch-1 and Notch-3 NICD were expressed in various carcinogenic breast gland cellular lines enhancing the results of Callahan and Raafat [77, 78]. Breast tumorigenesis is a consequence of abnormal genetic types of the Notch-4 NICD [54, 55]. In another clinical report, Notch-4 affected positively to a great extent the transforming potential of the mammary gland epithelial cell lines [51, 73].

The overexpressed type of Notch-2 receptor seems to be connected to improved patient clinical outcome; the presence of Notch-2 represents decreased tumor dissemination [51, 79].

Clinical investigation from Mittal et al. (2009) has provided us with evidence on the presence and expression of the Notch proteins (Notch-1, -2, and -4) and their cognate ligands (Jagged-1, Jagged-2, Delta-like ligand 1, and Delta-like ligand 4) in breast cancer in comparison to normal tissue. The pair receptor-ligand is present in significant numbers, proving that the notch system has an important role in breast carcinogenesis [43]. The Notch-3 receptor and Delta-like 1 ligand were undetectable in normal breast tissue; the same proteins were detected in high titers in a subset of breast tumors [80]. The interaction of the Notch and the Ras/MAPK signaling pathways has been detected in this clinical work as well as in other series [81].

Stylianou et al. (2006) investigated the action of the Numb gene (Notch inhibitor) on Notch signaling, particularly on Notch-1 and Notch-4 subtypes. The Numb proteins are encoded by the Numb gene and play important roles in cell's predetermined biochemical pathway; Numb is the negative regulator within the Notch vascular regulatory system in breast tumors. The precise action of Notch signaling and the vigorous expression of Notch-1 and Jagged-1 mRNA concentrations are characteristic features in breast neoplasia [42]. Rendering of Notch signaling to an active state has been established in a variety of neoplasms. Reedijk et al. (2005) observed that both Jagged-1 and Notch-1 receptors were predominantly expressed in breast tumors; these tumors demonstrated pathological characteristics, reduced overall survival, and poor prognosis due to the cooperative expression effect of the two receptors in breast tissue [44]. Dickson et al. showed that, in a sample of 127 breast cancer subjects, the increased Jagged-1 mRNA was a characteristic of patients with poor clinical outcome [68]. The possible existence of an activation loop has been suggested between the two receptors inducing tumor formation and spreading. It is highly probable that Notch signaling intermingles with tumor growth and diverts normal cells to an aberrant proliferative cellular pathway, that is, potential targets of this abnormal cellular diversion constitute a group of breast cancers, including EGF receptor- (EGFR-) positive, HER2/erbB2-positive, p53-negative, and estrogen receptor- (ER-) positive and negative cell lines. In these breast cancer cell lineage groups, clusters of the NICD component were detected; in particular, Numb upregulation was vanished in the same tumor samples; the Notch intracellular domain (NICD) is a reliable indicator of increased Notch signaling. The predominant site for Notch-1 receptor is the luminal area of normal breast cells, indicating the major role in cells' final destination. The detection of aberrant Notch signaling has been identified as positive on lobular and ductal breast norms. Aberrant Notch signaling expression seems to participate in both preinvasive ductal carcinoma in situ and metastatic mammary neoplasm, whereas the number of the overexpressed Notch receptors was increased [41, 82–84].

Clinical studies by Jubb et al. showed that lactating breast tissue demonstrated increased titers of Dll-4, one of the components of the Notch system; the same expression profile was observed in the malignant endothelium of invasive breast cancer and the angiosarcomas [83]. The vessels in the invasive breast cancer seem to be seized with the Notch ligand Dll-4. The Dll-4 expression profile in lactating mammary gland was equivalent to breast neoplasm [84, 85].

Different clinical reports focus on Notch-1 and its critical role in breast tumor evolvement; extremely aggressive cell lines, MCF-7 and MDA-MB231, express Notch-1 in high levels (Bolos et al. (2007) and Rizzo et al. (2008)). Clinical experimentation work on normal breast tissue and ductal carcinoma in situ (DCIS) revealed the expression of Notch-1 and Notch-4 receptors in the tumor tissues [85]. Notch-1 receptor was found in mammary tumors with the concurrent upregulation of H-ras. Breast cancer cell lines, MCF-7 and T47D, with decreased Jagged-2 activity promoted the caspase activity, a fact suggesting that Notch signaling prevents these cells from programmed cell death. Callahan et al. experimented on mice mammary tumor virus-induced neoplasms and identified Notch-4 to be the potent oncogene; Notch-4 changes the mammary epithelium towards the carcinogenic type [43, 49].

## 8. Cross-Talk between Notch and Estrogen Signaling Pathway

Estrogens are regulators of normal breast tissue considering growth and differentiation and have a major participation in ER(+) breast carcinoma [86]. As mentioned, the Notch pathway targets transcriptional repressors of the Hes and Hey subfamilies. Hes-1 of the Hey subfamily acts as a mediator of E2 estrogen (17*β*-estradiol).

Cross-talk between estradiol and Notch signaling has a major role in human breast carcinogenesis and angiogenesis. The interactive effect between estradiol and Notch signaling in breast cancer cells and endothelial cells was first described in 2004 by Soares et al. The authors reported that E2 promotes 8-fold increase in Notch-1 and 6-fold increase in JAG-1 expression in MCF-7 breast cancer cells. Similar regulation was found in endothelial cells for both Notch-1 and Jag-1 [87]. In 2008, Calaf and Roy reported that an organophosphate compound (parathion) and E2 either alone or combined led to activation of Notch pathway in MCF-10F [88]. Moreover, in the same year, Rizzo et al. presented a study, where authors reported that estradiol decreases Notch transcriptional activity in breast cancer cells via an estrogen receptor (ER) *α* [89], while in the presence or absence of estradiol, Notch-1 activates ER*α*-dependent transcriptions [90]. Therefore, estradiol regulates Notch signaling and Notch signaling regulates estrogen signaling in breast cancer cells, and combinations of antiestrogens and Notch inhibitors could be more effective in ER*α*(+) breast cancers [89].

## 9. Potential Therapeutic Inhibition of the Notch Signaling Pathway

Notch signaling has an undisputable participation in tumor vessel creation and particularly Jagged-1 and Dll-4 are crucial ligands for this procedure affecting both cancer cells and neighboring components. Jagged-1 has been proven to participate in multiple aspects of cancer biology including neoplastic cell growth and the metastatic process, promoting cancer stem cells and resistance to therapy, while Dll-4 has been proven to participate in the main procedure of tumor angiogenesis [91].

The connection between breast cancer and the Notch pathway was first reported by Jhappan et al. in 1992 when the mouse mammary tumor virus (MMTV) was inserted to modify the expression of the Notch-4 genes. In addition, in 1996, truncated Int3 (Notch-4) was expressed under the control of Whey Acidic Protein (WAP) promoter, with both studies finally reporting a formation of mammary carcinoma with subsequent lung metastasis due to abnormal mammary gland development [48, 55]. Notch-1 and -4 genes were also studied in 2004, as targets for insertion and rearrangement by the same virus (MMTV) that promoted epithelial mammary tumorigenesis [49, 92]. Moreover, in 2010, 296 breast adenocarcinomas and 38 ductal carcinoma in situ tissues were examined by Jubb et al. The study resulted in a Dll-4 expression associated with breast cancer cells, where Dll-4 was expressed by intratumoral cells in 73% to 100% of breast adenocarcinomas and 18% of in situ ductal carcinomas [83].

The connection between ligand blockage and potential therapeutic usage was inevitable, especially when evidence revealed overexpression of Jagged-1 and Dll-4 on breast tumor xenografts [83, 91].

To date, many scientists have investigated different inhibitors for aberrant Notch activation. Jagged-1 deletion caused deficits in vascular smooth muscle and fatal vascular defects, inhibiting sprouting angiogenesis, while Dll-4 blockade inhibits tumor growth, resulting in increased but dysfunctional angiogenesis, with other studies complementing an increased efficacy on the adjuvant chemotherapy when combined to anti-Dll-4 factors. Hoey et al. studied in 2009 Dll-4 antibodies and reported that Dll-4 inhibition either alone or combined to chemotherapeutic agent irinotecan reduced cancer cells [91, 93–95].

More recently, monoclonal antibodies inhibiting or recognizing specific ligands or receptors were developed. Notch-1, -2, and -3 receptors are tested and inhibited successfully by antibodies in experimental trials. The antibodies either block the connection of ligand to receptor or prevent expose of Kuzbanian or TACE cleavage site [93–95]. The first antibodies aimed at *γ*-secretase. Inhibition of *γ*-secretase prevented the cleavage of NEXT (a substrate cleaved by *γ*-secretase) and the release of NICD to the nucleus [96, 97]. Other *γ*-secretase inhibitors, the SAHM1 and the TR4, can inhibit all Notch receptors by preventing the formation of RBPjk/NICD/MAML transcriptional activator complex (they prevent MAML from binding to RBPjk/NICD), and both are antibodies that also seem to be well tolerated [98, 99]. Another *γ*-secretase inhibitor, Z-Leu-Leu-Nle-CHO, was proven to cause cell death even at minimum concentrations inhibiting proteosomal function unlike DAPT (GSI IX) and L685,458 (GSI X) inhibitors [100]. In 2016, Mamaeva et al. reported that nanoparticles loaded with a *γ*-secretase inhibitor to target Notch prevent promotion of cancer stem cells and tumor expansion [101].

However, many studies proved disadvantages of prolonged, and by extension, single therapeutic approach. Long-term treatment with Notch inhibitors was associated with vascular tumors, while many studies claimed that notch inhibition with the absence of additional therapeutic means fails to promote apoptosis [42, 102–104]. On the contrary, combination of Notch inhibitors and current therapies seems to increase effectiveness by resensitizing ER(+) and Her2(+) breast cancers to antiestrogen and anti-HER2 therapies, giving the most desired outcome [64, 89, 105–108]. These findings suggest that even though Notch inhibition seems very promising, well-designed clinical trials with integrated biomarkers are still both necessary and inevitable in order to develop effective molecularly targeted therapies.

## 10. Notch and Breast Cancer Stem Cells

Breast cancers contain cell populations, hierarchically organized, at the top of which are cells that display stem cell properties. Three theories have been proposed for the origin of Breast Cancer Stem Cells (BCSCs): (a) transformation of dormant normal stem cells to cancer stem cells via unsuitable regulation or mutations, (b) “misplacement somatic stem cell” theory, and (c) intratumoral lineages that have differentiated from common progenitor cells [109–111].

BCSCs have been identified from the CD44+/CD24- phenotype [112, 113]. CD44, glycoprotein in cell surface, is an element for breast cancer adhesion, migration, and invasion and leads to tumor progression via the interaction with osteopontin [114, 115]. Another glycoprotein also located in cell surface, CD24, participates in tumor growth and metastasis [116]. Moreover, ALDH1, the dominant form in the family of ALDH involved in the conversion of retinaldehyde to retinoic acid, is a recognized marker [117]. The role of BCSCs in cancer formation and progression is remarkable because, via the enhanced membrane transport, the DNA repair machine, the ROS scavenging systems, and the ability to detoxify cytotoxic drugs, BCSCs mediate metastasis and contribute to treatment resistance [118].

Three pathways seem to play key role in BCSCs formation, the Notch, the Hedgehog, and the Wnt, with Notch and Hedgehog being involved in self-renewal and differentiation of normal stem cell and result in a BCSC phenotype [119, 120].

Notch and HER2 pathways, both involved in regulation of cancer stem cells, reveal several interactions. Notch binding sequences are located in HER2 promoter and HER2 overexpressing cells demonstrate activated Notch signaling, while use of *γ*-secretase inhibitors (or a small interfering RNA) to block Notch expression results in downregulation of HER2 expression and decreased sphere formation [121, 122]. The role of Notch pathway is proven to be important in BCSCs and many scientists focused on clarifying this relation over the years [46, 82, 108, 123–126]. Notch-upregulated genes are located in breast cancer initiating populations called mammospheres (a clump of mammary gland cells that proliferate in spherical structures). Dontu et al. studied the effect of Notch activating in stem cells and proved that activation of Notch with DSL increased the number of multipotent cells, while on the contrary, using Notch-4 blocking antibody or GSI to inhibit Notch pathway abolished secondary mammosphere formation [46, 124]. Similarly, Farnie et al. studied the involvement of Notch in stem cells of breast ductal carcinoma in situ (DCIS) by examining accumulation of NICD and Notch-4 intracellular domain and the expression of Hes1 in DCIS tissue. An EGFR tyrosine kinase inhibitor (gefitinib), a *γ*-secretase inhibitor (DAPT), a goat anti-human Notch-4-neutralizing polyclonal antibody (clone-N17 without sodium azide), and a goat immunoglobulin G were added to DCIS cells suspension at time of plating and the mammospheres were counted on day 3 after plating. The levels of NICD and Hes1 were elevated in all samples, suggesting that breast cancer may be promoted by dysregulation of Notch signaling and that Notch-4 is directly involved in the regulation of DCIS mammosphere formation and/or growth [82]. Harrison et al., both in 2010 and 2013, also supported that self-renewal of BCSC is regulated by Notch signaling and added that Notch-4 knockdown has a more significant impact than Notch1 in BCSC and that Notch inhibitors operate downstream of oestrogen in the regulation of ER-negative cancer stem cells by blocking their activity and reducing their frequency [125, 126].

## 11. Conclusion

Breast cancer research has concentrated for a long period time on deciphering the hub of the mechanism of the neoplastic changes that occur on the epithelial tissue of the breast gland. In mammals, there is clinical and laboratory evidence that the Notch family is clearly implicated in human breast cancer in different combinations. The Notch pathway masters and maintains a balance between cell multiplication, differentiation, and programmed cell death, but the misregulated Notch ligand-receptor interaction in breast cancer gives the spark for tumor growth initiation, progression, and maintenance by inducing aberrant tumorigenesis, while determining vascular integrity. This is achieved through the “tip and stalk” cell selection procedure and act of the Notch-1, -4, and Dll-4 proteins that are all profoundly involved in the development of breast cancer.

Future clinical studies should aim at retrieving the entire pattern of the Notch family members engaging in different types of breast cancer and also need to provide more answers considering the potential therapeutic use of monoclonal antibodies inhibiting the Notch pathway, which already seems be effective, opening way for innovative treatments that, combined to current therapies, would overtake the obstacle of therapeutic resistance.

## Figures and Tables

**Table 1:** Breast cancer subtypes and expression of biological markers.

**Breast cancer subtypes**		**Immunohistochemical analysis**
**1**	**Luminal A**	PR(+) and/or ER(+) and HER2(-),↓ Ki67
**2**	**Luminal B**	PR(+) and/or ER(+) and HER2(+/-), ↑ Ki67
**3**	**Basal Like**	PR(-) and ER(-) and HER2(-)
**4**	**HER2 enriched**	PR(-) and ER(-) and HER2(+)
